# Fast calculation software for modified Look-Locker inversion recovery (MOLLI) T1 mapping

**DOI:** 10.1186/s12880-021-00558-8

**Published:** 2021-02-12

**Authors:** Yoon-Chul Kim, Khu Rai Kim, Hyelee Lee, Yeon Hyeon Choe

**Affiliations:** 1 Clinical Research Institute, Samsung Medical Center, Sungkyunkwan University School of Medicine, Seoul, South Korea; 2 Department of Electronic Engineering, Sogang University, Seoul, South Korea; 3 Department of Mathematics, Sogang University, Seoul, South Korea; 4 Department of Radiology and HVSI Imaging Center, Heart Vascular Stroke Institute, Samsung Medical Center, Sungkyunkwan University School of Medicine, 81 Ilwon-ro, Gangnam-gu, Seoul, 06351 South Korea

**Keywords:** MRI, Heart, T1 mapping, Parameter estimation

## Abstract

**Background:**

The purpose of this study was to develop a software tool and evaluate different T1 map calculation methods in terms of computation time in cardiac magnetic resonance imaging.

**Methods:**

The modified Look-Locker inversion recovery (MOLLI) sequence was used to acquire multiple inversion time (TI) images for pre- and post-contrast T1 mapping. The T1 map calculation involved pixel-wise curve fitting based on the T1 relaxation model. A variety of methods were evaluated using data from 30 subjects for computational efficiency: MRmap, python Levenberg–Marquardt (LM), python reduced-dimension (RD) non-linear least square, C++ single- and multi-core LM, and C++ single- and multi-core RD.

**Results:**

Median (interquartile range) computation time was 126 s (98–141) for the publicly available software MRmap, 261 s (249–282) for python LM, 77 s (74–80) for python RD, 3.4 s (3.1–3.6) for C++ multi-core LM, and 1.9 s (1.9–2.0) for C++ multi-core RD. The fastest C++ multi-core RD and the publicly available MRmap showed good agreement of myocardial T1 values, resulting in 95% Bland–Altman limits of agreement of (− 0.83 to 0.58 ms) and (− 6.57 to 7.36 ms) with mean differences of − 0.13 ms and 0.39 ms, for the pre- and post-contrast, respectively.

**Conclusion:**

The C++ multi-core RD was the fastest method on a regular eight-core personal computer for pre- or post-contrast T1 map calculation. The presented software tool (fT1fit) facilitated rapid T1 map and extracellular volume fraction map calculations.

## Background

Cardiac T1 mapping in magnetic resonance imaging (MRI) is a non-invasive and quantitative method for the characterization of the myocardial tissue [1–4] and is particularly useful for the evaluation of diffuse myocardial fibrosis [5]. It typically involves two separate image acquisitions: native T1 mapping (a.k.a. pre-contrast T1 mapping) and post-contrast T1 mapping. Extracellular volume fraction (ECV), which is a biomarker for myocardial fibrosis, can be attained in a pixel-wise manner from the pre- and post-contrast T1 maps [6, 7]. Due to its quantitative nature, cardiac T1 mapping is advantageous over late gadolinium enhanced imaging, in which the accurate nulling of the healthy myocardial signals in an inversion recovery sequence is challenging in patients with diffuse myocardial fibrosis. The pattern of diffuse myocardial fibrosis is typically observed in patients with non-ischemic heart disease, such as hypertrophic cardiomyopathy (HCM), cardiac amyloidosis, and dilated cardiomyopathy [5].

T1 map calculation involves curve fitting for the quantification of T1 longitudinal relaxation time on a pixel-wise basis. The curve fitting process is time-consuming in general, and low-level programming languages such as C and C++ are desirable for improved computational efficiency. T1 parameters are estimated via non-linear least squares, and Levenberg–Marquardt (LM) optimization is typically utilized with good initial values of the parameters in the T1 fitting model. Alternatively, to overcome the issue of initialization of the parameters and to reduce the search space, Barral et al. presented a reduced dimension non-linear least squares (RD-NLS) approach, which resulted in initialization-free optimization and acceleration in T1 map calculation [8]. In other studies, T1 map calculation was reported to take longer than a minute per image [9, 10]. Recent related studies of software development in parameter mapping focused on magnetization transfer imaging [11] and neuroimage processing [12], and they lack the comparison of computational efficiency among different calculation methods.

The existence of a variety of methods for T1 map calculation motivated us to develop a software tool for evaluating the performance of the methods. In particular, we sought to develop a Python-based user interface that can also test a C++ implementation with pybind11 [13]. The interface setup facilitates the comparison between Python-based and C++-based methods. Moreover, the Python language serves as a framework for deep learning libraries [14–16] and is commonly adopted for the development of deep learning algorithms, which may have potential for improving the performance in cardiac T1 mapping [17]. In an earlier study, we demonstrated fast T1 map calculation using the LM-based method implemented in C++, as a module for a comprehensive quantitative cardiac MRI analysis tool [18]. In the present study, we focus on evaluating the performance of different T1 map calculation methods with an emphasis on the comparison between the LM method and the RD-NLS method implemented in C++ as well as an emphasis on the comparison between the RD-NLS method and the publicly available MRmap for T1 estimation accuracy in the myocardium.

## Methods

**Fig. 1:**
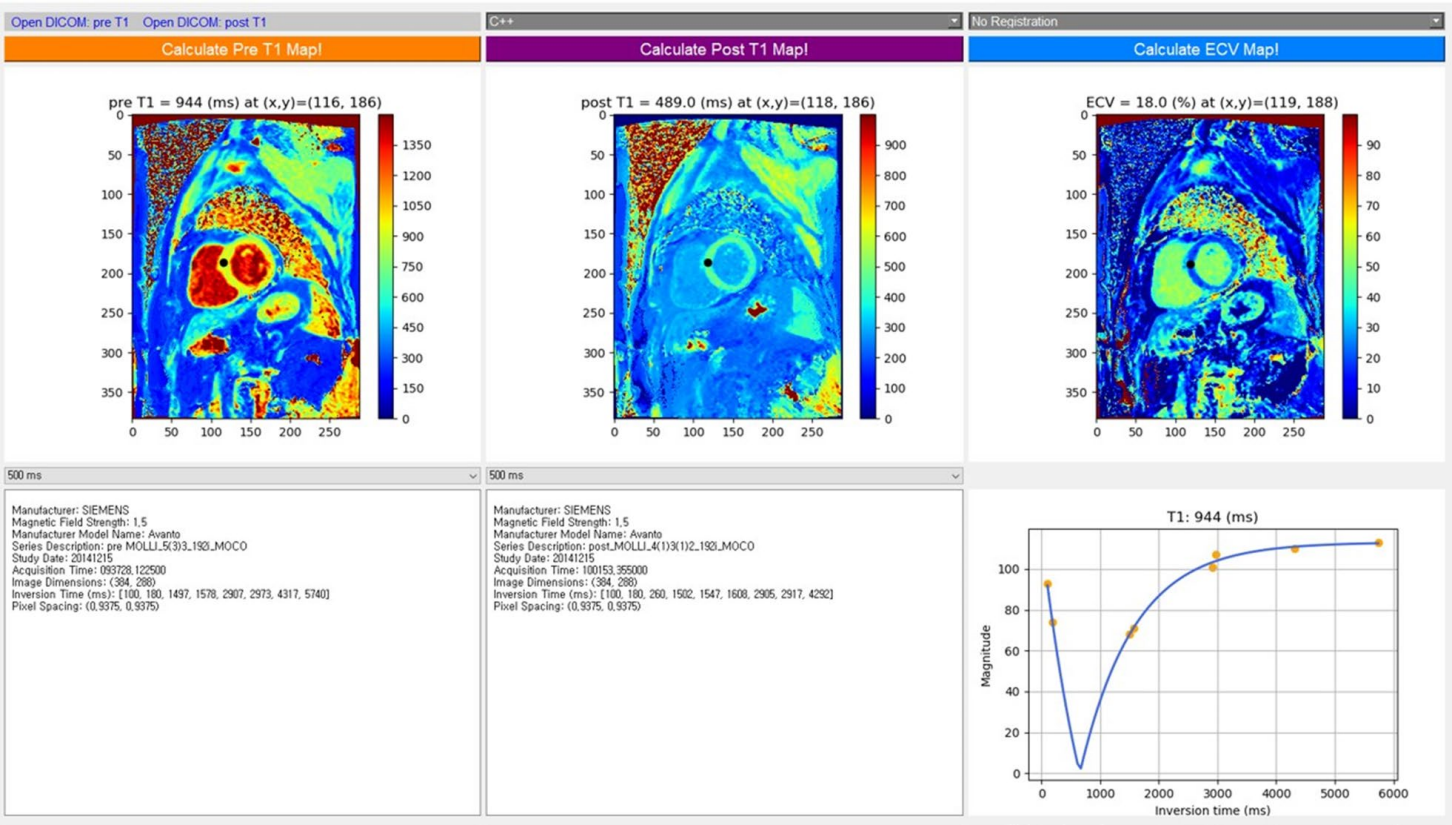
A screenshot of the user interface for the calculation of pre- and post-T1 maps as well as an ECV map. The interface allows a user to select a T1 map calculation method for the comparison of computation time and accuracy. It also provides ECV map calculation as well as T1 fitting result in a user interactive way

We describe the T1 mapping sequence parameters, T1 map calculation methods, and their implementations and evaluations. Figure 1 shows a custom user interface tool for the study, which is available at https://sites.google.com/site/yoonckim1/software/t1_map_compare. Source code is available at https://github.com/prime52/fT1fit.

### Data acquisition

Cardiac MRI scans were performed on a 1.5 T scanner (Siemens Avanto, Erlangen, Germany). Clinical MR examinations were approved by our institutional review board, and informed consent was obtained from the subjects prior to MRI scans. Subjects with suspected cardiovascular diseases were enrolled between May 2014 and May 2015. Subjects who took cardiac pre- and post-contrast T1 mapping scan exams were included for the present study. Subjects with inadequate image quality due to severe motion were excluded. A total of 30 subjects were considered for our study. They consisted of 9 HCM patients, 10 cardiac amyloidosis patients, 7 coronary artery disease patients, and 4 healthy volunteers.

The modified Look-Locker inversion recovery (MOLLI) sequence was used for cardiac T1 mapping [19, 20]. Imaging parameters were slice thickness = 8 mm, echo time (TE) = 1.01 ms, spacing between slices = 20 mm, the number of phase encoding steps = 104, pixel bandwidth = 1085 Hz, acquisition matrix = 192 × 120, image matrix = 384 × 288, pixel spacing = 0.9375 mm × 0.9375 mm, and field of view (FOV) = 360 mm × 270 mm. The MOLLI protocols used were different for the pre-contrast and post-contrast T1 mapping. The MOLLI 5(3)3 protocol used for pre-contrast T1 mapping consisted of 5 inversion time (TI) image acquisitions after the first inversion pulse, a three-heartbeat pause for the recovery of the longitudinal magnetization, and 3 TI image acquisitions after the second inversion pulse. The MOLLI 4(1)3(1)2 protocol used for post-contrast T1 mapping consisted of 4 TI image acquisitions after the first inversion pulse, a one-heartbeat pause, 3 TI image acquisitions after the second inversion, a one-heartbeat pause, and 2 TI image acquisitions after the third inversion. Since the post-contrast T1 relaxation time is approximately less than 500 ms, which is much shorter than the pre-contrast T1 (~ 950 ms for myocardium and ~ 1500 ms for blood), the 3 inversion pulses used in the 4(1)3(1)2 protocol enables more adequate sampling of the early part of T1 relaxation than the 2 inversion pulses used in the 5(3)3 protocol. Instead of 5 images after the first inversion, 4 images were acquired in the post-contrast T1 mapping because for the short T1 (< 500 ms) relaxation the image from the fifth heartbeat appears similar to the image from the fourth heart beat due to fast T1 recovery and thus is unnecessary [21]. The TI images were acquired in a diastolic cardiac phase. All of the TI images were aligned using the motion correction algorithm [22]. The TI images were exported as digital imaging and communications in medicine (DICOM) files for T1 map calculation.

### T1 Map calculation

T1 map calculation was performed pixel-by-pixel. In general, the TI images can be available as either complex-valued or magnitude-valued. In the present study, the DICOM dataset was available as magnitude TI images, and most cardiac MRI scanner systems in hospitals save the DICOM dataset in the magnitude scale by default. At each voxel’s location (x, y), the signal intensity $S(t)$ can be modeled as the following T1 relaxation curve.

1 $$S\left(t\right)=a\left(1-{e}^{-bt}\right)+c$$

For a set of inversion times *t* = [${TI}_{1}$*, *${TI}_{2}$*, …,* ${TI}_{N}$] and a set of corresponding signals [${S(TI}_{1}),{S(TI}_{2}), \dots , {S(TI}_{N})$], there are *N* equations and 3 unknowns, which are $a$, *b*, and *c*. In the present study, *N* was 8 for the pre-contrast data, while *N* was 9 for the post-contrast data. Notably, *b* is the reciprocal of T1* (i.e., the apparent T1). The objective function $f$ to be minimized was then given as the sum of squares of the difference between the relaxation model and the data.

2 $$f(a,b,c)=\sum_{i=1}^{N}{\left(S\left({TI}_{i}\right)-a(1-{e}^{-b{TI}_{i}}\right)-c)}^{2}$$

This is a nonlinear least squares problem, and the Levenberg–Marquardt (LM) algorithm [23] can be used to iteratively estimate the parameters $a$, *b*, and *c*. We empirically selected the initial values for the parameter set ($a$, *b*, *c*) = (350, 0.001, − 150) for the pre-contrast data and (350, 0.005, − 150) for the post-contrast data. Since our dataset was in the magnitude scale and $S(t)$ of Eq. (1) can cover the negative-value range, we incrementally flipped the polarity of $S({TI}_{i})$ to find the best fit of Eq. (1) [24]. Notably, the estimated T1* does not incorporate the effect of the tip down of the spin magnetization for every repetition time. Hence, the Look-Locker correction was applied in the following way to arrive at the corrected T1 value:

3 $$T1={T1}^{*}\left(\frac{a}{a+c}-1\right)$$

Meanwhile, the RD-NLS (or RD) method expands the objective function Eq. (2) and finds the optimal estimates of the parameters separately (refer to Appendix B in Barral et al. [8] for detailed mathematical derivations). The function to be optimized for T1 is independent of the other two parameters. Hence, the decoupling leads to a one-dimensional search problem for T1 estimation.

### Python and C++ implementation

**Fig. 2:**
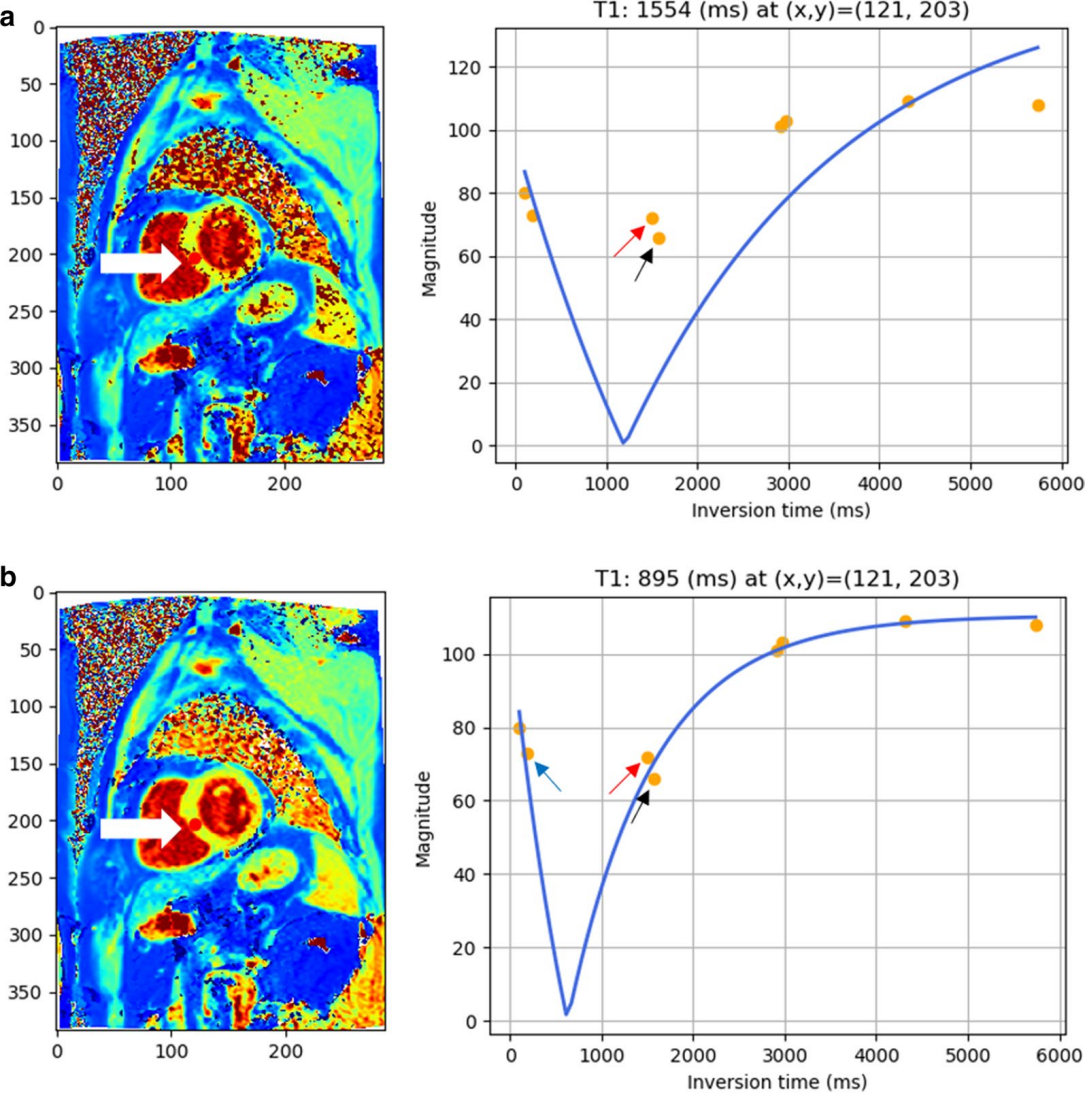
An example of improved fitting result after modification of the original RD-NLS method. The unsatisfactory performance of the original RD-NLS occurred when some inversion time (TI) values were very close such that the choice of minimal TI closest to 0 can be sensitive to noise. **a** A poor fitting result when the RD-NLS method was applied without any modification of the script. **b** A correction after the modification, which considered three candidate samples for polarity restorations (see arrows)

For the LM method, we implemented the T1 map calculation in Python. To solve the non-linear least squares problem, we used the scipy.optimize.curve_fit() function, which is based on the Levenberg–Marquardt algorithm. The method is referred to as LM_python. For the RD method, we converted the original MATLAB script written by Barral et al. (code available at http://www-mrsrl.stanford.edu/~jbarral/t1map.html) to Python. We noted that the original RD algorithm failed to work in some cases where the two candidate TIs produced unsatisfactory fitting results. Hence, we modified the RD algorithm by introducing three candidate TIs. This helped remove the unsatisfactory fitting and improved the accuracy (Fig. 2). The method is referred to as RD_python.

We also implemented both the LM- and RD-based T1 map calculations in C++ and used pybind11 [13] to make the compiled C++ code compatible with the Python environment. The C++ implementation was performed on a Windows OS, Microsoft Visual Studio 2017 platform. For the LM-based T1 parameter estimation, we used the solve_least_squares_lm function of the Dlib library [25]. In addition, for the multi-core implementation, we used the OpenMP library [26] for the parallelization of the ‘for’ loop in the pixel-wise T1 map calculation. We chose the static schedule option for parallelization. For evaluation, the methods are referred to as LM_C++_single-core, RD_C++_single-core, LM_C++_multi-core, and RD_C++_multi-core.

To ensure that the curve fitting is correctly performed at each pixel location and the samples are placed close to the T1 relaxation curve, we developed a graphical user interface (GUI) that enables a user to load the DICOM TI image data, select a method for T1 map calculation, and mouse-click a pixel location for displaying its curve fitting result. The GUI also performs the calculation and display of the pre- and post-contrast T1 maps as well as the ECV map (Fig. 1). The GUI was implemented using the PyQT library [27] on a 64-bit Windows PC.

### Evaluation

We evaluated the following methods in terms of speed on a Windows PC (AMD Ryzen 7 1800X Eight-Core Processor and 16.0 GB RAM): MRmap, LM_python, RD_python, LM_C++_single-core, RD_C++_single-core, LM_C++_multi-core, and RD_C++_multi-core.

For the evaluation, we used the MRmap software [9], which is publicly available for download at https://sourceforge.net/projects/mrmap/. We chose the following options for T1 map calculation: Limits of T1, T2, and Noise for pre-contrast were set to 3000, 350, and 0, respectively. Limits of T1, T2, and Noise for post-contrast were set to 1500, 350, and 0, respectively. Specifically, the setting of the Noise value had to be consistent, since the choice of the value significantly affected the computation speed. Registration was set to None. Process was set to “T1 mapping—MOLLI,” and Correction was set to “Look-Locker.”

For the evaluation of accuracy in T1 value, we manually drew a region of interest (ROI) in the myocardium in either a pre- or a post-contrast T1 map of each subject and used the same ROI mask for the T1 maps estimated using MRmap and the RD_C++_multi-core method. Mean T1 values were calculated within the myocardial ROIs. Bland–Altman analysis was performed by computing the mean difference and 95% limits of agreement between the two T1 measurements.

## Results

**Fig. 3:**
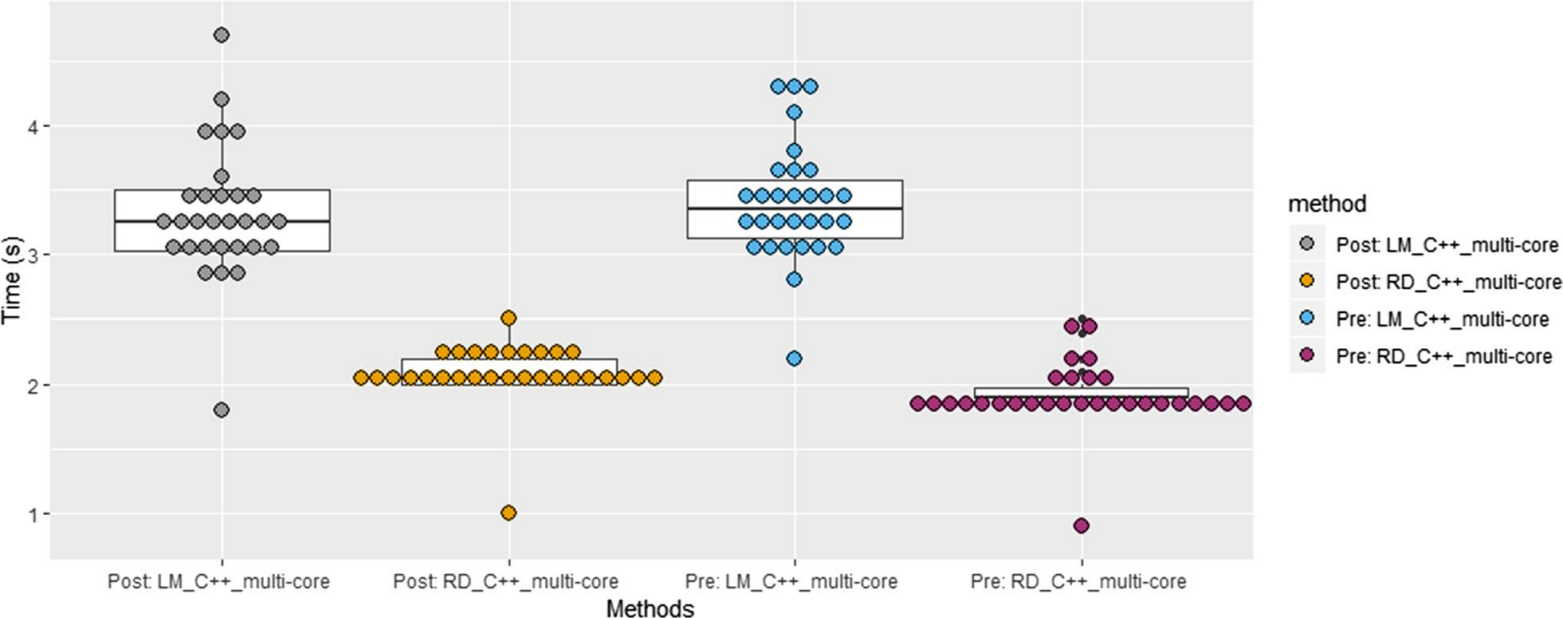
Comparison of computation time. RD_C++_multi-core took shorter computation time than LM_C++_multi-core in both pre- and post-contrast cases. The outliers (i.e., the lowest value in all four methods) took the shortest computation time due to the smallest image dimensions (256 × 218), while most of the images are of the dimensions (384 × 288)

Table 1 lists the computation time for MRmap, LM_python, RD_python, LM_C++_single-core, RD_C++_single-core, LM_C++_multi-core, and RD_C++_multi-core for pre- and post-contrast TI image sets in 30 subjects. The RD method was superior to the LM method in computation time for all three different ways of implementation: python, single-core C++, and multi-core C++. The RD_C++_multi-core method took approximately 2 s for T1 map generation in both pre- and post-contrast T1 maps. There were statistically significant differences in computation time between RD_C++_multi-core and LM_C++_multi-core: 1.9 s vs. 3.4 s (p < 0.001) for pre-contrast and 2.1 s vs. 3.3 s (p < 0.001) for post-contrast. The distributions of computation time clearly exhibit the superior performance of RD_C++_multi-core (Fig. 3).

**Table 1:** Comparison of computation time in the pre- and post-contrast T1 map calculations (n = 30). The measurements in the columns indicate median (interquartile range) expressed in seconds

Method	Pre-contrast	Post-contrast
MRmap	126.0 (98.3–140.6)	111.8 (93.7–140.6)
LM_python	261.3 (249.3–282.4)	249.6 (242.6–262.1)
RD_python	77.0 (74.0–80.1)	77.8 (75.9–81.4)
LM_C++_single-core	28.6 (27.3–29.8)	28.0 (26.9–29.9)
RD_C++_single-core	15.2 (15.0–15.9)	16.2 (16.1–16.8)
LM_C++_multi-core*	3.4 (3.1–3.6)	3.3 (3.0–3.5)
RD_C++_multi-core*	1.9 (1.9–2.0)	2.1 (2.0–2.2)

^*^Eight cores were simultaneously used for the calculation

**Fig. 4:**
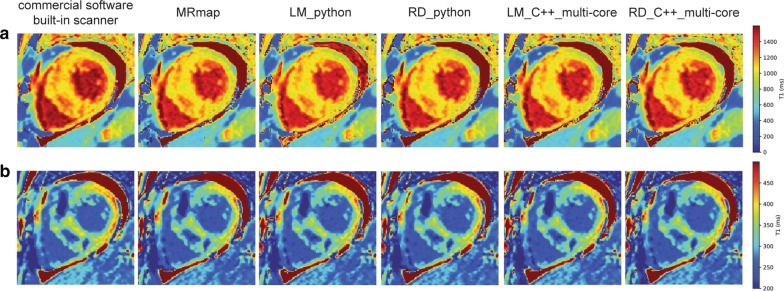
Qualitative comparison of T1 mapping accuracy in a subject with hypertrophic cardiomyopathy. **a** Pre-contrast T1 maps. **b** Post-contrast T1 maps. All the methods resulted in similar T1 measurements in the myocardium and blood

**Fig. 5:**
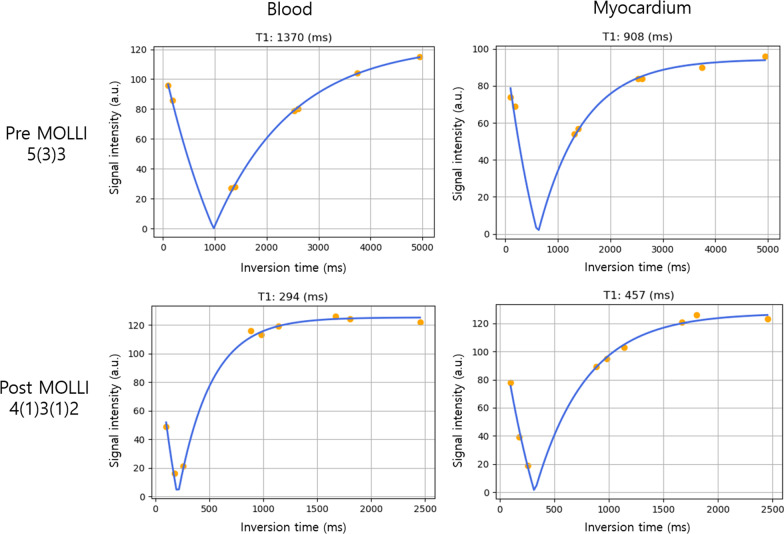
Curve fitting examples when the RD_C++_multi-core method was used. (Top row) curve fits of the samples acquired using the pre-contrast MOLLI 5(3)3 protocol. (Bottom row) curve fits of the samples acquired using the post-contrast MOLLI 4(1)3(1)2 protocol. The left plots represent curve fitting on a voxel corresponding to the left ventricular blood pool, while the right plots represent curve fitting on a voxel corresponding to the myocardium. The solid blue line denotes the estimated T1 relaxation curve after fitting. Note that the nulling time typically ranges 200–400 ms for the post-contrast, and acquiring three samples instead of two in early TIs in post MOLLI is helpful in estimating short T1 values, which are typical in post-contrast tissue

Qualitative comparisons of the methods in pre- and post-contrast T1 measurements are shown in an HCM patient (Fig. 4), and all the methods produced similar T1 measurements in the myocardium and blood regions of interest. These similarities were also observed in other subjects’ T1 map data. Curve fitting examples for the pre-contrast and post-contrast MOLLI protocols are shown for the blood and myocardium in a normal volunteer (Fig. 5). In particular, the post-contrast MOLLI 4(1)3(1)2 protocol, which uses three inversion pulses, is advantageous in estimating a short T1 value by fitting three samples in early TIs, over the pre-contrast MOLLI 5(3)3 protocol, which uses two inversion pulses.

**Fig. 6:**
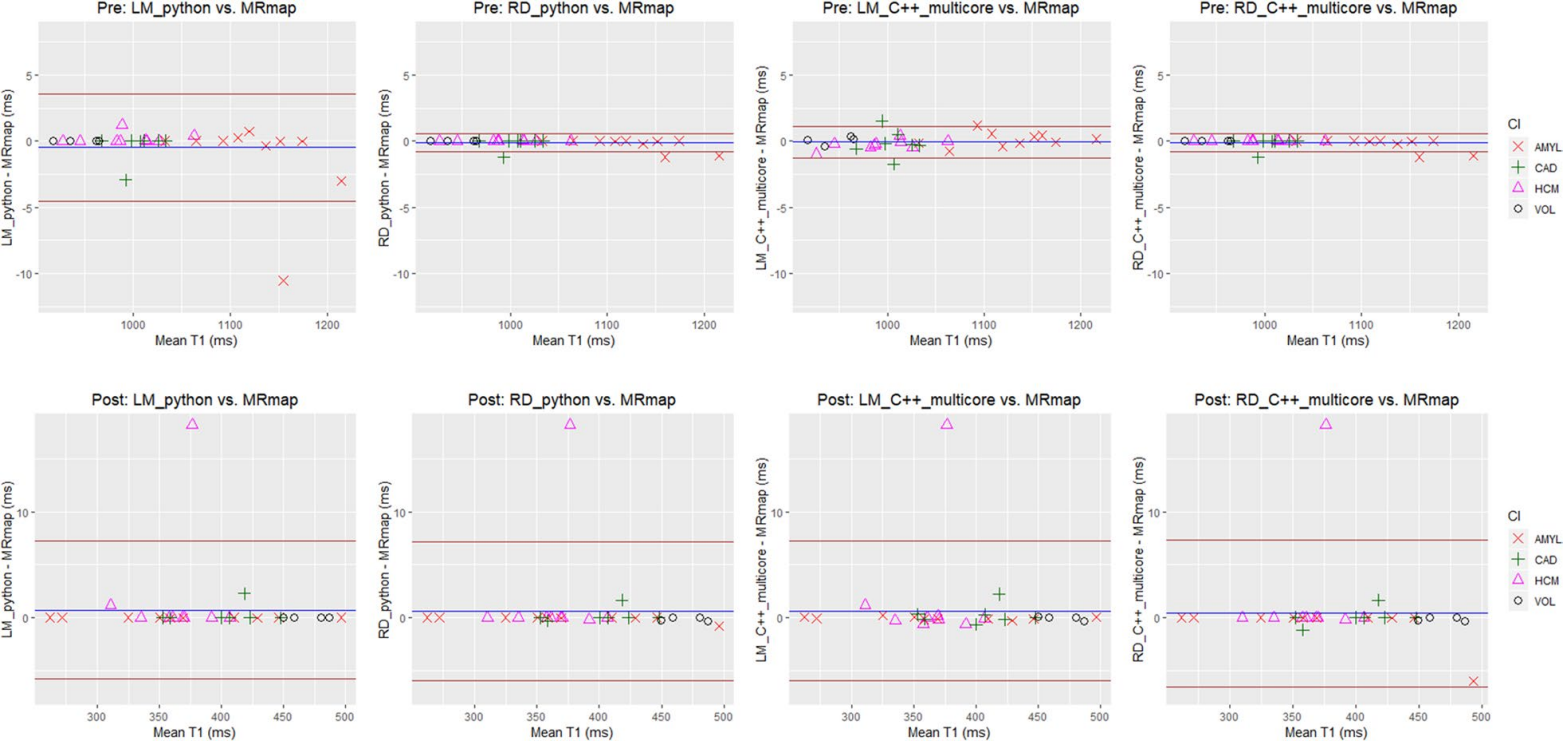
Bland–Altman plots for the four T1 calculation methods when compared with MRmap as reference. (Top row) pre-contrast T1 measurements in the myocardium. (Bottom row) post-contrast T1 measurements in the myocardium. From left to right, (1) LM_python, (2) RD_python, (3) LM_C++_multi-core, and (4) RD_C++_multi-core

T1 measurements in the myocardium for the four methods (i.e., LM_python, RD_python, LM_C++, RD_C++) are shown in Fig. 6 for the pre-contrast (top row) and post-contrast (bottom row) cases. The Bland–Altman plots show good agreement between each of the four methods and the reference MRmap in all subjects except for a few outliers. Most of the T1 difference values lie within 2 ms. Table 2 shows Bland–Altman statistics for the four methods. For all the methods, the absolute mean difference was small (< 1 ms) for the pre- and post-contrast cases. The 95% limit of agreement was wider for the post-contrast than for the pre-contrast, but this is likely due to the outlier whose difference value was approximately 18 ms (see the plots in the bottom row of Fig. 6). As expected, the amyloidosis group (AMYL) shows high pre-contrast T1 values in the myocardium (see the plots in the top row of Fig. 6).

**Table 2:** Bland–Altman statistics for the myocardial T1 measurements with MRmap as the reference method

	Pre-contrast		Post-contrast	
	Mean Difference, ms	95% Limits of Agreement, ms	Mean Difference, ms	95% Limits of Agreement, ms
LM_python	− 0.24	− 5.11 to 4.63	0.72	− 5.81 to 7.25
RD_python	− 0.13	− 0.83 to 0.58	0.60	− 5.95 to 7.14
LM_C++_multi-core*	− 0.04	− 1.34 to 1.25	0.63	− 5.96 to 7.22
RD_C++_multi-core*	− 0.13	− 0.83 to 0.58	0.39	− 6.57 to 7.36

^*^T1 measurements between multi-core and single-core were the same

## Discussion

We demonstrated a rapid T1 map calculation method, which only took approximately 2 s for 384 × 288 × 8 or 384 × 288 × 9 images on a personal desktop computer equipped with an eight-core processor. This is a significant improvement over other methods demonstrated in the literature. For comparison, we measured computation time on MRmap, which was 126 s in pre-contrast and 112 s in post-contrast. In the literature, MRmap was reported to take 113 s for a set of 128 × 128 × 8 images with a noise level of 0 [9]. The MRmap code was written in Interactive Data Language (IDL), which is a high-level language so that it is computationally slow. Other software tools also deserve to be mentioned. The T1 map calculation of Altabella et al. was reported to take 66 s for the magnitude fitting on 31,080 fitted voxels from a set of 218 × 256 × 8 images [10]. The T1 map calculation of Liu et al., referred to as the vectorized Levenberg–Marquardt fitting, was reported to take 60 s on average in MATLAB for a set of 256 × 256 × 8 images. It is undeniable that the C++ nature of the proposed method was the main cause of the speed improvement. However, it is important to note that the way of implementation via the Python wrapper facilitated code readability and maintenance. It is noted that a C++ implementation for T1 mapping was recently demonstrated by Werys et al. [28], although we did not compare their method with our methods.

There may be room for improvement in computational speed in the proposed method. First, the noise level (or the threshold level) can be applied to the images as was demonstrated in [9, 10]. By increasing the threshold up to an acceptable level, one can exclude a fairly large number of pixels whose intensity is not sufficiently high (e.g., the background air region) from the curve fitting process, and this could help reduce the computation time. Second, loop scheduling can be optimized when using the OpenMP library. In general, OpenMP provides three kinds of scheduling: static, dynamic, and guided. There may be an opportunity for speed improvement by, for example, trying these scheduling methods with different choices of chunk size in dynamic mode and finding the optimal one.

Python provides a convenient environment for developers in terms of code readability and thus facilitates the debugging process. This was especially true in the design of a T1 map calculation algorithm and a graphical user interface in the present study. We first implemented a Python version of the T1 map calculation and translated it to a C++ version. For the verification of the C++ implementation in terms of accuracy, we compared the T1 maps generated by the Python implementation and the C++ method within the graphical user interface. This was helpful in evaluating the accuracy of the C++ implementation.

In this study, different MOLLI sequence protocols were used in the acquisitions of pre-contrast and post-contrast T1 data: MOLLI 5(3)3 for the pre-contrast and MOLLI 4(1)3(1)2 for the post-contrast. There are other MOLLI sequence variants reported in the literature (Table 2 of Kellman and Hansen [20]). Intra-individual comparisons among the MOLLI sampling schemes may be worth investigating because the curve fitting on different sampling schemes may affect the T1 map results as well as the ECV map results. However, implementing the intra-individual comparisons would be challenging due to patient discomfort as a result of repeated use of contrast agent and repeated breath-holds.

The rapid computation framework based on pybind11 and multicore C++ has the potential to be applied to three-dimensional (3D) T1 mapping [29], which has larger dimensions than 2D T1 mapping, as well as to other pixel-wise parameter estimation of T2, T2*, tissue perfusion, and permeability. For example, deconvolution process in model-based tissue perfusion quantification [30] can be accelerated using C++ and parallel processors.

The current study has several limitations. First, this was a single-center study conducted using data acquired on the Siemens 1.5 T scanner, with a small number of subjects (n = 30) for evaluation. Second, the tool supported only the magnitude data rather than both magnitude and complex-valued data. Third, the computation time was assessed on a single desktop computer only. Evaluation on other types of computers (e.g., mobile phone with a restricted capacity for central processing unit (CPU) and memory, workstation computer with a higher capacity for CPU and memory) would be worth investigating.

## Conclusions

We evaluated the performance of the RD-NLS in terms of speed and accuracy compared with that of the LM method. The RD-NLS implemented with C++ and parallel processing library was the fastest, taking < 3 s in both pre- and post-contrast T1 map calculation on a regular desktop computer. The Python wrapper has the potential to improve workflow in the implementation of rapid pixelwise parametric mapping, not merely of T1 estimation but also of tissue perfusion- and permeability-related parameter estimation. The implementation details available as open source may be helpful resources for other researchers’ investigations and validation of new methods in parameter mapping.

## Data Availability

Data in the present study are not publicly available since they include private patient information. The de-identified data are available from the corresponding author upon request after the approval of the institutional review board of Samsung Medical Center. The code is available at https://github.com/prime52/fT1fit.

## Abbreviations

MRI: Magnetic resonance imaging
ECV: Extracellular volume fraction
LM: Levenberg–Marquardt
RD-NLS: Reduced dimension non-linear least squares
MOLLI: Modified Look-Locker inversion recovery
TE: Echo time
FOV: Field of view
TI: Inversion time
DICOM: Digital imaging and communications in medicine
GUI: Graphical user interface
ROI: Region of interest
HCM: Hypertrophic cardiomyopathy
AMYL: Amyloidosis
CAD: Coronary artery disease
VOL: Healthy volunteer
CPU: Central processing unit
